# Socio-demographic and political predictors of Theory of Mind in adulthood

**DOI:** 10.1371/journal.pone.0284960

**Published:** 2023-05-24

**Authors:** Rachel A. Clutterbuck, Mitchell J. Callan, Punit Shah

**Affiliations:** Department of Psychology, University of Bath, Bath, United Kingdom; University of Bergamo, ITALY

## Abstract

Individual differences in Theory of Mind (ToM)–the ability to understand the mental states of others–are theorised to be predicted by socio-demographic and political factors. However, inconsistent findings on the relationships between various socio-demographic predictors and ToM, as well as a paucity of research on political predictors of ToM, have left a gap in the literature. Using a recently validated self-report measure of ToM in a large sample (*N* = 4202) we investigated the unique contributions of age, sex, socio-economic status, and political beliefs to ToM in adults. Except for age, all variables were correlated with ToM, but when accounting for the variance of other predictors in statistical analyses, political beliefs was no longer associated with ToM. Dominance analysis revealed that participant sex was the most important predictor of ToM. These findings help to address theoretical discrepancies in the existing literature and inform future methods and directions in social cognition research.

## Introduction

Theory of Mind (ToM) is the ability to attribute non-emotional mental states, such as beliefs, perceptions, and intentions, to oneself and others (e.g., [1]). It is an important social ability that enables us to socially interact and form relationships with others [2]. Measuring ToM, however, is not straightforward. Issues in the validity, reliability, and practical utility of existing measures (e.g., [3,4]), as well as differences in conceptualisations of ToM (e.g., [5]) have hampered ToM research designs, sample sizes, and the validity of some existing findings. ToM measures also do not typically correlate well with each other (e.g., [6]) suggesting they may be measuring divergent constructs. Together, these issues have generated many inconsistent results and misunderstandings within the ToM literature.

One example of such inconsistencies is found in relation to age and ToM in adulthood. Age is known to be an important predictor of ToM in childhood (e.g., [7]), during the development of social and cognitive abilities, and also in late adulthood [8], potentially through associations with general age-related cognitive decline [9]. There are, however, inconsistent findings on the association between age and ToM across the adult lifespan, with studies sometimes finding no relationship (e.g., η_p_^2^ = .02; [10]) or a negative relationship between age and ToM (e.g., η_p_^2^ = .11 [11]; cf. [12]). (*F* converted to η_p_^2^ using effectsize package in R (v. 0.8.3) [13]. Effect size represents a ToM ‘bias’ index, with positive directionality indicating age is associated with poorer ToM performance.) There are several potential reasons for such inconsistent results. For example, Duval et al. found that effects of age-related decline in ToM ability across the lifespan was present for some ToM tasks, but not others [14]. Given that affective and cognitive social abilities have shown differential age-related effects in adulthood (e.g., [15]), varying degrees of emotional content in the ToM measures may contribute to inconsistent findings. As well as this, the different additional cognitive demands of some behavioural ToM measures (e.g., on executive functions) may sometimes disadvantage older people [16], causing sporadic and misrepresented effects of age-related ToM decline in adulthood. Most studies have also only explored categorical differences in ToM between age groups, rather than continuous relationships between age and ToM [8,9] which could reveal more nuanced changes in ToM across the lifespan. For example, the current evidence of a positive association between age and ToM in early life (e.g., [7,17]), and a negative association in later life (e.g., [8]), potentially suggests a quadratic relationship between age and ToM across the lifespan. Additionally, research posits that inconsistent findings of age differences in ToM may be explained by different levels of education between older and younger participants in the samples (e.g., [18]). Clarifying the typical developmental trajectory of ToM in adulthood is important for identifying atypical development, as observed in various adult-onset conditions (e.g., neurodegenerative diseases; [19]).

The relationship between ToM and socio-economic status (SES) is similarly poorly understood. While some research has found that higher SES predicts poorer ToM (e.g., (*r* = -.25, [20]), others have found the inverse relationship, whereby higher SES predicts greater ToM (e.g., β = .23 –.90; [21]). Meta-analyses exploring this relationship in child samples have found a modest positive relationship between SES and ToM (*r* = .18; [22]). Other studies have found no association between SES and ToM (e.g., *r* = -.10 –-.12; [23]). Exploring this issue, Deveney et al. found that the two main indices of SES–income and education–had differential relationships with ToM; higher income was associated with better ToM, but education was not significantly associated with ToM, and these results were different in smaller samples [24]. Deveney et al.’s findings suggest that inconsistent findings on the relationship between ToM and SES may be driven by the use of different indices of SES (education and income). A critical issue with these and previous findings, however, is that the ToM measures used may not actually be measuring ToM, but rather empathy or emotion processing (e.g., [3]), casting doubt on the results. Further research is therefore needed, using a well-validated measure of ToM, to understand the relationship between different indicators of SES and ToM.

Inconsistencies in the ToM literature are likely accentuated by i) differences in statistical power to detect effects due to (in)sufficient sample sizes, and ii) the use of different ToM measures between studies, which are potentially measuring different social-cognitive processes instead of ToM (e.g., [3,6]). To help address this issue, a questionnaire measure of ToM has been developed (Four-Item Mentalising Index (FIMI); [25]), which overcomes longstanding concerns with the validity, reliability, and practical utility of classical ToM measures. Critically, the FIMI measures the ability to understand *non-emotional* mental states, in line with contemporary conceptualisations of ToM (e.g., [26]) and therefore does not conflate cognitive and affective social-cognitive processes. The FIMI can be used to address outstanding questions and inconsistencies within the ToM literature by collecting large samples of online ToM data, enabling even small effects to be identified through well-powered statistical analyses. Using this measure, in a large sample, we aimed to examine relationships between ToM and socio-demographic predictors which have shown inconsistent associations with ToM (e.g., age, socio-economic status).

The first aim of the current research was to clarify the nature of the relationships between socio-demographic predictors and ToM, and to investigate their *unique* associations with ToM, by controlling the variance of all other predictors included in the statistical models. We do this to ensure that relationships between each predictor and the criterion variable are not confounded by the effects of other predictors. To this end, sex was also included in the statistical models. Sex and ToM have often been associated, with women consistently outperforming men in childhood (e.g., [7]) and adulthood (e.g., [25]). As yet, there is no definitive explanation for this ToM sex difference, but it could be due to various biological mechanisms and social factors relating to sex equality (e.g., [27]). Sex has also been found to moderate the relationship between age and various cognitive functions (e.g., [28]), including ToM. For example, in older populations, women process ToM stimuli more slowly than men ([29]). It is therefore important that sex is included in analyses involving ToM to ensure that sex differences in ToM do not influence the findings of the study. Recent research has also claimed there are *not* sex differences in ToM among adults (e.g., [30,31]). Therefore, by including sex in our analyses, the current study can clarify previous findings on sex and ToM using a psychometrically robust measure of ToM, while controlling other socio-demographic and political predictors of ToM, in a uniquely large sample.

The current study additionally aims to explore a potentially novel predictor of ToM, political beliefs, which, is also theoretically associated with the aforementioned socio-demographic factors (e.g., SES; [32], age [33], sex [34]). Political beliefs are known to be associated with numerous cognitive factors, such as attentional biases [35], and social-cognitive processes. Liberalism, for example, has been positively associated with greater social connectedness [36], compassion [37], and empathy [38]. Such associations between political beliefs and social-cognitive processes may have interesting, motivational underpinnings, whereby liberals may be more motivated to understand the emotions of others due to their egalitarian beliefs [38]. Yet, to our knowledge, there have been no direct investigations into the relationship between political beliefs and ToM. Exploring this further may speak to a better understanding of the perceived “Empathy Gap” in political ideology–the idea that liberals tend to be more caring of others than conservatives (e.g., [39])–alongside providing a less confounded examination of the link between socio-demographic factors and ToM.

Overall, the current research will quantify the unique contributions of i) sex, ii) income, iii) political beliefs, iv) age, and v) education to ToM. Based on the existing literature outlined in this Introduction, we expect that i) women, ii) having a higher income, and iii) liberal beliefs will be associated with greater ToM, and iv) age will be negatively associated with ToM. v) We expect to find support for the null hypothesis on the relationship between education and ToM, in line with the recent literature.

## Materials and methods

### Participants, measures, and procedure

A large, convenience sample of 4202 adults (3021 women, 1172 men, 9 neither man nor woman) aged 18–99 years (*M*_*age*_ = 52.37, *SD*_*age*_ = 14.78) from the UK were recruited via several online sources, including being directed to the survey through news articles and social media. Participants whose geo-location fell outside of the UK were excluded (*n* = 2,372), according to the UK-specific measures used in the study. Using a within-subjects design, this sample provided us with >99% power to detect very small associations between the predictor variables and ToM (*f*^*2*^ = .01).

After written informed consent was obtained, participants reported their age, sex (assigned at birth), education (eight-point scale with categories from 0 = no qualifications, to 7 = PhD/DPhil/level 8 diploma; [40]), number of adults and children living in their household, and annual household income (eighteen categories from 1 < £5,000, to 18 > £85,001). Annual household income responses were recorded as the category mid-points, and the value for the open-ended upper category was calculated using a pareto curve estimator, as shown in Callan et al. [41]. Adjusted income was calculated to indicate spending power, using the formula seen in Skylark and Baron-Cohen [42] which divides annual household income by (number of adults + 0.5 x number of children). Political beliefs were measured using a 7-point Likert scale from 1 = very liberal, to 7 = very conservative, a scale which has been used in previous research (e.g., [43]).

ToM was measured using the Four-Item Mentalising Index (FIMI; [25]), in which participants answered four questions, including “*I find it easy to put myself in somebody else’s shoes*”, on a four-point Likert scale from 1 = strongly disagree, to 4 = strongly agree, generating a total score between 4 and 16. The FIMI has previously shown good internal consistency, test re-test reliability, a unifactorial structure, and associations with a cognitive ToM measure and a measure of autistic traits [25].

The study was provided written approval by the University of Bath, Department of Psychology ethics committee and conducted in line with the 1964 Declaration of Helsinki and its later amendments.

### Statistical analyses

Alpha was set at .05 for all frequentist statistics. Given the large sample size, null hypothesis significance tests were sensitive to small effects. We therefore accompanied frequentist statistics with Bayesian analyses to provide an indication of the strength of the evidence, and Bayes Factors (BFs) were interpreted using the adjusted guidelines reported in Wagenmakers et al. [44], whereby BF_10_ > 3 provides substantial support for the alternative hypothesis, and BF_10_ < 1 provides support for the null hypothesis. All analyses tested two-tailed hypotheses. Analyses were performed using JASP v. 0.12.2.0.

Participants who reported their sex as *neither man nor woman* were not included in analyses involving sex (*n* = 9), and 123 participants that did not report their education level could not be included in analyses involving education.

## Results

Descriptive and reliability statistics are reported in Table 1. Sex, education, political beliefs, and adjusted income were significantly correlated with ToM. More specifically, education and income were both positively associated with greater ToM. Liberalism was associated with greater ToM, and women reported higher ToM than men. However, Bayesian analyses suggested only anecdotal support for the correlation between adjusted income and ToM. The correlation between age and ToM was not significant, with Bayesian analyses indicating evidence for the null hypothesis on this relationship (Table 2).

**Table 1:** Participant characteristics and descriptive statistics.

	Women	Men	Total
*M* age (years)	51.29(14.55)	55.07(14.87)	52.37(14.78)
Education (%)			
No qualifications	9.69	7.89	9.19
GCSE or equivalent	23.68	18.58	22.25
A-Level or equivalent	16.96	12.71	15.77
Level 4 NVQ or equivalent	4.88	5.17	4.96
Level 5 NVQ or equivalent	5.56	6.66	5.87
Bachelor’s degree or equivalent	18.56	23.05	19.82
Master’s degree or equivalent	16.45	17.44	16.72
PhD or equivalent	4.23	8.50	5.43
*M* political beliefs	3.82(1.51)	4.06(1.70)	3.89(1.57)
*Median* political beliefs	4	4	4
*M* adjusted income (£)	20,130.10 (18,151.04)	26,845.79 (22,585.64)	21,993.48 (19,713.05)
Theory of Mind (FIMI score)	13.15(2.19)	12.40(2.44)	12.94(2.29)

*Note*. *N* = 4202 (3021 women, 1172 men, 9 neither man nor woman). Standard deviation in parentheses. The FIMI showed acceptable-to-good internal consistency (greatest lower bound = .72; *ω* = .68).

**Table 2:** Correlations among socio-demographic predictors, political beliefs, and ToM.

Measure	1	2	3	4	5	6
1. Age	–					
2. Sex	.12**^	–				
3. Education	.13**^	.10**^	–			
4. Adjusted income (£)	.12**^	.15**^	.37**^	–		
5. Political beliefs	.10**^	.07**^	-.23**^	.03^†^	–	
6. Theory of Mind	-.00^†^	-.15**^	.08**^	.05*	-.05**^	–

*Note*. Pearson’s *r* correlations are reported, with point-biserial correlations for binary variables.

*p < .05,

**p < .001,

^BF_10_ > 3,

^†^BF_10_ < 1.

Sex was coded as Men = 1, Women = 0.

Multiple regression analysis confirmed that sex, education, and adjusted income uniquely predicted ToM, but age and political beliefs did not predict ToM (*F* [5, 4066] = 27.21, *p*< .001, *R*^*2*^ = .03; Table 3). Averaging across models, Bayesian modelling revealed that the data provided strong support for sex and education predicting ToM, and anecdotal support for adjusted income predicting ToM. Comparatively there was moderate-to-strong evidence against age and political beliefs predicting ToM (Table 3).

**Table 3:** Multiple regression and dominance analyses of socio-demographic predictors of ToM.

Predictor	*B*	*SE B*	*β*	*t*	*p*	*sr* ^*2*^	95% BCa CI		BF_inclusion_	GDW
							Lower	Upper		
Age	0.00	0.00	.00	0.16	.875	.00	-0.00	0.01	0.06	.000
Sex	-0.81	0.08	-.16	-10.04	< .001	.02	-0.97	-0.64	∞	.023
Education	0.07	0.02	.07	4.09	< .001	.00	0.04	0.11	1869.78	.005
Adjusted income (£)	0.00	0.00	.05	2.87	.004	.00	0.00	0.00	2.51	.002
Political beliefs	-0.04	0.02	-.03	-1.62	.105	.00	-0.09	0.01	0.23	.002

*Note*. BF_inclusion_ = inclusion Bayes Factor (higher values indicate stronger support for inclusion of the predictor in the final model). GDW = General Dominance Weight (higher values indicate greater relative importance of the predictor). 95% bootstrapped bias-corrected and accelerated confidence intervals (95% BCa CI) with 10,000 resamples are reported. Bayesian analyses were performed using the JASP (v. 0.12.2.0) default priors. No multivariate outliers were identified in the multiple regression analysis (all Cook’s distance values < 1).

To test the relative importance of the predictors for ToM, we employed a dominance analysis using the yhat package [45] in R (v. 3.5.3). Dominance analysis compares the relative contribution of each predictor to the criterion variable across all possible sub-set regression models, to establish a dominance hierarchy between predictors (see [46]). A dominance analysis with age, sex, education, adjusted income, and political beliefs predicting ToM, was performed. Bootstrapping (10,000 resamples) estimated reproducibility rates (RR) reflecting how likely the observed dominance relationships would occur in the population, based upon how often it occurred in the bootstrapped samples. As reported in Table 3, the general dominance weights, which reflect the average contribution of each predictor across all possible subset models and always sum to the overall model *R*^*2*^, revealed that sex was the most important predictor of ToM (RR = 100%), followed by education (RR > 88%), then adjusted income and political beliefs as equally important predictors–comparison of the dominance relationship between adjusted income and political beliefs revealed similarly low reproducibility rates (adjusted income > political beliefs, RR = 68.3%; political beliefs > adjusted income, RR = 67.7%) suggesting a dominance hierarchy cannot be assumed between these two variables. Finally, age was the least important predictor of ToM (RR > 93%).

Previous findings of lower ToM in groups of adults with a mean age over 65 years (e.g., [8]) potentially suggests there could be a non-linear relationship between age and ToM across the lifespan. To explore a non-linear relationship between age and ToM, we regressed FIMI scores on participant age and the quadratic term for age (age^2^). Analyses revealed a significant quadratic relationship between age and ToM, *β* = -.44, *t*(2,4199) = -4.49, *p* < .001, with Bayesian analyses indicating strong support for the quadratic modelling of age predicting ToM (BF_inclusion_ = 40.90). The data suggests there is a negative relationship between age and ToM among participants 65+ (*n* = 991; *r* = -.11, *p* < .001, BF_10_ = 13.51), and a slightly positive relationship between age and ToM among participants younger than 65 (*n* = 3211; *r* = .05, *p* = .009, BF_10_ = 0.65), although Bayesian analyses suggest support for the null hypothesis for the relationship between age and ToM in the under 65’s age group.

## Discussion

As expected, sex and income significantly predicted ToM. Political beliefs initially correlated with ToM, but this relationship did not hold when other socio-demographic predictors were controlled in multiple regression analyses. Contrary to the previous literature, education *was*, but age *was not*, a significant predictor of ToM. When exclusively examining older age groups, age and ToM were associated. The most dominant predictor of ToM was sex, followed by education, then political beliefs and income, and finally age. The findings clarify discrepancies in the existing literature and inform future research methods.

In line with most previous literature (e.g., [7,25]), sex predicted ToM, with women reporting greater ToM than men. Sex was also the most dominant predictor of ToM, highlighting the importance of accounting for this variable in ToM research. The ToM measure used in this study, the FIMI, measures the same construct in men and women, and correlates with cognitive measures of ToM ([25]), allaying concerns that these results may be explained by sex differences in question interpretation or self-report biases. Whether this sex difference is driven by biological or environmental factors, or even personality differences (e.g., [47]) is currently unknown but a potentially interesting area for future research. This finding contradicts research suggesting there are no sex differences in ToM (e.g., [30,31]) and negates claims that sex differences can be explained by differences in education [30]. Failure to identify a sex difference in ToM in previous research may be due to smaller sample sizes (e.g., [30]), or controlling psychological factors such as anxiety (e.g., [31]), which influence social cognition, and vary between sexes (e.g., [48]).

The two markers of SES, income and education, were positively associated with ToM. This contradicts recent findings on this topic (e.g., [20]), but is consistent with research using similar SES indicators (e.g., [21]). Contrary to other large-cohort studies (see [24]), education was a positive predictor of ToM, and indeed ranked the second most dominant predictor of ToM. By operationalising education as a binary variable, Deveney et al. may have reduced their statistical power to detect associations between education and ToM [24]. In the current study, frequentist statistics suggested that income was significantly associated with ToM, but Bayesian analyses provided only anecdotal support for this association. Income was also an equally dominant predictor of ToM as political beliefs–a variable which was not associated with ToM in the multiple regression analyses. From these results, we tentatively suggest that income is a very weak predictor of ToM. This may explain why results on the relationship between income and ToM have varied between studies (see [24]). To our knowledge, the current study is the first large-cohort study to show that a positive relationship between SES and ToM remains after controlling for other demographic variables, likely because we had greater statistical power than previous studies to detect this result. There are several potential explanations for an association between higher SES and greater ToM, such as differences in executive functioning or verbal ability between high and low SES individuals, which require further investigation. For example, low SES has previously been associated with poorer executive functioning skills (e.g., [49]) and verbal ability (e.g., [50]), both of which are associated with ToM ability (e.g., [51]). Future investigations into whether the association between SES and ToM can be better understood as an impairment of several interrelated cognitive processes (e.g., ToM, executive functioning, verbal ability) in low SES individuals, could be informative towards understanding social class divisions, and developing interventions for low SES individuals.

Initially, liberal beliefs were associated with greater ToM, which is consistent with the existing, albeit limited, literature on political beliefs and social cognition. Critically, however, this relationship disappeared when controlling socio-demographic information in the analyses. Suggesting that the observed correlation between political beliefs and ToM was being driven by socio-demographic factors. This implies that previous findings of significant relationships between political beliefs and social cognition, which have not controlled for socio-demographic factors (e.g., [52]), may not represent reliable effects. Future research should therefore test the robustness of previous findings after controlling socio-demographic factors. There are, however, concerns about the reliability of using single-item political ideology measures, as these measures could be particularly influenced by self-report biases and may oversimplify political ideology. Therefore, re-examining this relationship using a multi-item measure of political ideology in future research would be necessary to establish the reliability of these findings. As liberals have been found to exaggerate their responses in relation to moral political matters ([43]), future research should also confirm these findings using non-self-report methods.

This is the first study to find support for the null hypothesis on the relationship between age and ToM when examining the entire adult lifespan. This clarifies discrepancies in the existing literature, and suggests that overall ToM is stable throughout adulthood. Exploratory analyses revealed that age and ToM were negatively correlated in the 65+ age group, which is consistent with previous literature suggesting age-related ToM decline in later life ([8]). This is the first study to show this association using a self-report measure of ToM, providing the first indication that older adults may have reliable insight into their own ToM decline. While this contradicts previous findings of no age-related effects on self-reported social cognition ([14]), this is likely due to the larger sample size and continuous conceptualisation of age in the current study, enabling more highly powered analyses to detect age-related effects. Further research investigating the reliability of this ToM insight in older adults by comparing self-reported ToM to performance on a cognitive measure of ToM could inform our understanding of metacognition in aging populations, and clarify the utility of self-report measures to understand age-related cognitive decline. Future studies could also investigate whether there are differences in response-style between older and younger individuals using self-report ToM measures. Results of age-related effects in the under 65s age group were less clear cut, with frequentist statistics suggesting a positive relationship between the variables but Bayesian analyses suggesting no relationship. As stated in our Methods section, due to the large sample, some frequentist statistics may be sensitive to small effects. So, Bayesian analyses may provide a more reliable indication of the nature of this relationship in the under 65s age group. Together, therefore, the results suggest that there were no age-related individual differences in self-reported ToM across the lifespan, but that a decline in ToM ability can be detected in adults over the age of 65 years. This informs understanding of ToM development and, enabled by the large sample and continuous conceptualisation of the constructs, provides a unique insight in the nuanced changes of ToM in late adulthood.

## Conclusion

The current research investigated the associations between several socio-demographic and political predictors with ToM. The results confirmed that sex, education, and income predicted ToM, but there was no linear association between age or political beliefs and ToM across the lifespan. The results add clarity to the existing literature, inform future ToM research methods of socio-demographic predictors that should be controlled in analyses, and inspire several future research directions to confirm and better understand the current findings.

## Supporting information

S1 Data (CSV)
